# Exercise-Induced Pulmonary Hypertension: A Valid Entity or Another Factor of Confusion?

**DOI:** 10.3390/life13010128

**Published:** 2023-01-03

**Authors:** Elina Khattab, Nikolaos Velidakis, Evaggelia Gkougkoudi, Nikolaos P.E. Kadoglou

**Affiliations:** Medical School, University of Cyprus, 2029 Nicosia, Cyprus

**Keywords:** exercise-induced pulmonary hypertension (EIPH), pathophysiology, right heart catheterization (RHC), exercise stress echocardiography (ESE), cardiopulmonary exercise testing (CPET)

## Abstract

Exercise-induced pulmonary hypertension EIPH has been defined as an increase in mean pulmonary arterial pressure (mPAP) during exercise in otherwise normal values at rest. EIPH reflects heart and/or lung dysfunction and may precede the development of manifest pulmonary hypertension (PH) in a proportion of patients. It is also associated with decreased life expectancy in patients with heart failure with reduced ejection fraction (HFrEF) or left ventricle (LV) valvular diseases. Diastolic dysfunction exacerbated during exercise relates to increased LV filling pressure and left atrial pressure (LAP). In this context backward, transmitted pressure alone or accompanied with backward blood flow promotes EIPH. The gold standard of EIPH assessment remains the right heart catheterization during exercise, which is an accurate but invasive method. Alternatively, non-invasive diagnostic modalities include exercise stress echocardiography (ESE) and cardiopulmonary exercise testing (CPET). Both diagnostic tests are performed under gradually increasing physical stress using treadmill and ergo-cycling protocols. Escalating workload during the exercise is analogous to the physiological response to real exercise. The results of the latter techniques show good correlation with invasive measurements, but they suffer from lack of validation and cut-off value determination. Although it is not officially recommended, there are accumulated data supporting the importance of EIPH diagnosis in the assessment of other mild/subclinical or probably fatal diseases in patients with latent PH or heart failure or LV valvular disease, respectively. Nevertheless, larger, prospective studies are required to ensure its role in clinical practice.

## 1. Introduction

Exercise-induced pulmonary hypertension (EIPH) has been defined as an exaggerated increase in mean pulmonary artery pressure (mPAP) >30 mmHg and total pulmonary resistance (TPR) > 3 Wood units during exercise, while an mPAP <25 mmHg is present at rest [1]. Patients are free of symptoms at rest, and they complain only of fatigue or dyspnea during exercise.

It is increasingly recognized that EIPH may represent an early, mild stage of pulmonary arterial hypertension, and preliminary data suggest its association with an overall higher morbidity and mortality [2]. Past evidence has shown the relationship between exercise-induced elevation of pulmonary artery pressure (PAP) with exercise intolerance and exertional dyspnea in patients without established diagnosis of pulmonary hypertensions (PH), underlining its clinical importance [3]. In addition to this, EIPH has been associated with reduced exercise capacity and worse functional status in patients with heart failure with either reduced ejection fraction (HFrEF) or preserved ejection fraction (HFpEF) [4] or other cardiac disorders (cardiac restriction, chronotropic incompetence) [5]. Furthermore, the early appearance of EIPH in patients with left ventricle (LV) valvular diseases, such as mitral regurgitation (primary or secondary), and mitral and aortic stenosis, may be of clinical relevance, giving a valuable tool for the effective risk stratification of asymptomatic patients. EIPH may be undiagnosed in a significant proportion of patients with exertional dyspnea but normal resting PAP because of the considerable limitations of PAP measurements during exercise. Right heart catheterization (RHC) during exercise seems to be the most objective approach for conclusive diagnosis, but it is invasive and sometimes complicated, limiting its clinical applicability. Other non-invasive, objective, reproducible and easily performed diagnostic methods of EIPH assessment are required in clinical practice but they have made the exact definition of EIPH a subject of debate using variable cut-off values (e.g., pulmonary artery systolic pressure (PASP)).

The term “exercise induced pulmonary hypertension” was suspended at the 4th World Symposium of Pulmonary Hypertension in Dana Point, in 2008, largely due to failures at reaching a consensus. Since then, a plethora of studies have been published about EIPH, its pathophysiology and methods of assessment. A body of data increasingly supports EIPH as an important and promising entity for the research and clinical community. Despite this, its unambiguous association with exertional dyspnea and its clinical usage in the prevention, early diagnosis and management of cardiopulmonary pathologies remains controversial. For all these reasons, we have conducted this literature narrative review to shed more light on the underlying mechanisms, the diagnostic methods and the potential clinical applications of EIPH.

## 2. Materials and Methods

For the needs of this narrative review paper, we searched MEDLINE and EMBASE, Web of Science, Cochrane and Google Scholar databases for English language publications from 1990 to May 2022. We also checked the reference lists of the identified articles to find any additional relevant articles. Our search included the titles, abstracts and medical subject headings (MeSH), and we used the following search terms or abbreviations: pulmonary hypertension, exercise-induced pulmonary hypertension (EIPH), pulmonary artery systolic pressure, pulmonary artery pressure, pathophysiology, right heart catheterization (RHC), exercise stress echocardiography (ESE), cardiopulmonary exercise testing (CPET), HFpEF, HFrEF, aortic stenosis, mitral stenosis and mitral regurgitation. Two investigators (N.V. and E.K.) performed the literature search independently.

In our search, we included clinical studies (both clinical investigations and meta-analyses) as well as experimental studies. To draw firm conclusions, we excluded the studies fulfilling the following criteria: unavailable full texts, publication language other than English and conference abstracts.

Using the abovementioned terms, we initially found 1457 hits. After the abstracts’ screening, we removed 126 duplicated studies and 1137 irrelevant studies; 194 full-text studies were screened for eligibility. After removing the studies with wrong design, irrelevant outcomes and unavailable full text, we ended up with a total of 78 studies, 3 systematic reviews and meta-analyses of clinical data and 30 additional clinical studies which were not included in those meta-analyses.

## 3. Pathophysiology of EIPH

Significant progress has been made in understanding the pathophysiology of the hemodynamic abnormalities of cardiopulmonary circulation during exercise. Although EIPH pathogenesis is still not completely understood, it can be attributed to alterations in pulmonary artery flow affected by cardiac output (CO), pulmonary vascular resistance (PVR) and left atrial pressures (LAP) during exercise, as indicated by the Poiseuille–Hagen equation: mPAP = PVR × CO + LAP [6]. EIPH appears to be most similar to the WHO group 2 PH [5], and the following 3 pathophysiological mechanisms have been proposed:

(A) Increased pulmonary flow: Unambiguously, cardiac output (CO) and, consequently, pulmonary blood flow significantly increase during exercise in response to the increased oxygen uptake. In parallel, there is a significant pulmonary vascular dilatation as a compensatory mechanism. The magnitude of the inevitable elevation of PAP and, to a lesser extent of wedged PAP (wPAP), is a product of increased blood flow balanced by pulmonary vascular resistance (PVR). In healthy individuals, PVR as a result of pulmonary vascular distensibility is widely adapted to high pulmonary blood flow in order to maintain PAP within a normal range [7]. Previous invasive assessments have shown that wPAP and left ventricular filling pressure (named end-diastolic LV pressure) can rise >30 mmHg during exercise in athletes and exercising elderly [8]. A disproportional increase in CO over moderately declined PVR results in high wedged PAP (wPAP) and LAP during exercise. A possible explanation for this derives from the Frank–Starling mechanism matching of the LV flow output to the peripheral demand [8]. Moreover, the elite athletes can generate extraordinarily high cardiac flows and exceed mPAP of 30 mmHg at very high levels of exercise. It has been hypothesized that the additional effect of pericardial constrain, in particular, the increasing LV diastolic volume, competes for space with the right ventricle within the relatively non-distensible pericardium. In that case, the LV diastolic compliance is possibly impaired resulting in a remarkable elevation of LAP [7].

**Figure 1:**
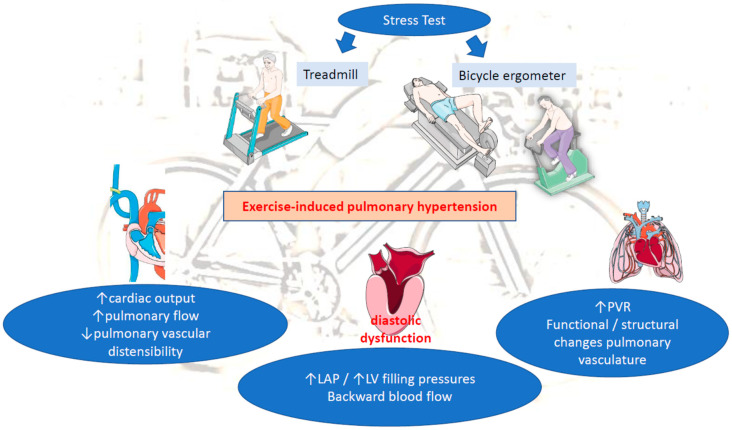
Pathophysiology of EIPH. LAP: left atrial pressure, LV: left ventricular, PVR: pulmonary vascular resistance.

Another factor determining pulmonary blood flow and PAP elevation during exercise is the type of exercise. During aerobic exercise, pulmonary blood flow has shown a linear progressive increase and thus a linear adjustment in the mPAP–O correlation may occur, whereas resistance exercise is associated with exaggerated pulmonary vasoconstriction and higher PVR [3,6]. The fact that mPAP is a flow-dependent variable, which can reach “pathologic” values in subjects without cardiopulmonary diseases, remarkably confuses the definition of EIPH.

(B) Increased LAP: Evidence supports the relationship between EIPH and increased LAP as a result of left cardiac disorders, such as advanced diastolic dysfunction or severe mitral valvular diseases [9]. Notably, high LAP may induce an early rise in pulmonary artery systolic pressure (PASP) at early stage of the exercise test [9]. A retrograde transmission of high LAP to the pulmonary arteries explains most of the rise in mPAP [7]. Hence, EIPH appears to be associated with increased LV filling pressure upon exertion, in the context of significant diastolic dysfunction (grade II or III) [10,11,12]. In addition, increased LAP due to LV failure, aortic stenosis or mitral stenosis (MS) may result in elevated mPAP on exertion [3]. Beyond the transmission of augmented LV filling and LAP, the elevation of wPAP may also indicate exercise-induced volume overload due to mitral regurgitation (MR) [7]. Backward blood flow from the LA to pulmonary circulation leads to pulmonary congestion and further PAP increase during exercise, underlying the contribution of dynamic MR on the development of EIPH [13].

(C) Increased pulmonary vascular resistance (PVR): Another determinant of mPAP is PVR. Normally, an increase in CO and LAP, during exercise, distends the pulmonary circulation, leading to decreased vascular resistance [3,9]. When both disorders are present (increased LAP and PVR), this implicates a mixed cardiopulmonary disorder often observed in patients with chronic LV diseases (HFrEF, mitral disease, etc.). A prolong exposure of pulmonary circulation to repeated elevations in PAP in those patients may lead to initially functional/reversible and afterwards structural restriction of pulmonary vascular distensibility and thereby to progressive impairment of pulmonary vascular adaptation to exercise-induced pulmonary flow. This will end up in time causing permanent changes in the pulmonary vasculature and the development of PH at rest. The loss of pulmonary vascular distensibility alone results in poor exercise adaptation and reflects lung pathology (e.g., chronic obstructive pulmonary disease (COPD), interstitial lung disease) [3,10,14,15]. Therefore, EIPH without concomitant abnormal elevation in LAP more likely reflects early pulmonary vascular disease, rather than pathology of the left heart (e.g., heart failure, valvular diseases, etc.) (Figure 1).

Overall, EIPH includes precapillary PH with abnormal pulmonary circulation and post-capillary PH with early rise in wPAP [16]. The occurrence of EIPH is present in a wide spectrum of left heart diseases, LV diastolic dysfunction, pulmonary vascular diseases, COPD, auto-immune diseases (e.g., scleroderma) and often a combination of those entities [17,18]. Furthermore, EIPH could be a potential marker for the risk of PH establishment in patients with connective tissue disorders [10]. Additional experimental and clinical studies are important for a better understanding of EIPH’s underlying pathology and interplay with nosology.

## 4. Methods of EIPH Assessment and Limitations

Currently there is not a consensus regarding the ideal way of detecting EIPH. Diagnostic modalities include ESE, CPET and RHC during exercise. Up until now, the latter has been considered the gold standard method for EIPH. However, there is no widely accepted strategy for EIPH diagnosis and thereby established presence.

### 4.1. Exercise Stress Echocardiography (ESE)

ESE seems to be a useful tool for the diagnosis of EIPH. The test is performed under gradually increasing physical stress, and different protocols have been proposed [19]. The Bruce protocol is performed in an upright position, using a standard treadmill setup until the targeted heart rate is achieved or symptoms’ appearance prevents the continuation of the test [20]. Alternatively, ergo-cycling in a semisupine position is preferred. In ergo-cycling, the most popular protocol is the WHO one, starting at 25 Watts and then followed by increases of 25 Watts every 2 min, again until the targeted heart rate is achieved, or symptoms arise. Sometimes, protocol adjustments may be applied according to a patient’s physical condition and previous experience. For example, a light ergo-cycle protocol with a starting workload of 10 W and incremental workload of 10 W every 2 min may be preferred in older patients. The duration of an ergo-cycle exercise test is wise to range between 6 and 10 min, depicting a good exercise capacity [21]. A shorter or longer duration of the test, without previous adjustment of the workload, may lead to the cessation of the test due to leg fatigue without achieving the target heart rate, leading to inconclusive results.

Using treadmill patterns of exercise, all echocardiography-related measurements are conducted just before the start for the treadmill protocol and immediately after the cessation of walking on the treadmill. That time, the patients should be transferred from the standing to the supine position on a coach, always next to the treadmill device, in order to obtain the transthoracic echocardiographic measurements as soon as possible (within 30 s). This is very challenging because pulmonary vascular pressures and flows return rapidly to baseline resting state after the completion of exercise [3]. A great advantage of the ergo-cycling over the treadmill protocol is the acquisition of images and measurements during the exercise test, without interrupting the participant’s exercise. On the other hand, the treadmill protocol offers more flexibility in exercise testing because it is the “natural” way of exercise, and less coordination from the subjects is required. Non-experienced cyclists may struggle to maintain a constant repetition rate, or they may experience muscle fatigue of the upper leg at the early stages of cycling, which may lead to the premature cessation of the test, interfering with its results.

Another important limitation of ESE regarding the diagnosis of EIPH is the need for trained and experienced operators to get accurate and reproducible results. Respiratory motions of the chest during exercise may prevent the acquisition of optimum echocardiographic images or the loss of the tricuspid regurgitant (TR) blood flow jet. The latter is a prerequisite for TRVmax and PASP calculations. A study compared mPAP measurements derived from ESE with measurements using exercise cardiac MRI and concomitant invasive pressure registration. This showed a strong correlation between non-invasive and invasive measurements, although non-invasive ESE tended to overestimate mPAP [22]. Some studies have been conducted assessing EIPH while individuals were doing a handgrip at 40% of the maximum voluntary contraction for 3 min or arm adduction lifting of dumbbells. However, this mode of exercise has substantial inferiority for EIPH evaluation during cycling [23].

Finally, ESE provides the PASP values and the ratio of early diastolic transmitral flow velocity to early diastolic mitral annular velocity (E/e’). Both of them can be easily calculated compared to the CPET parameters [24] and have been incorporated in the diagnostic algorithm of HFpEF in patients with “unexplained dyspnea” [25]. The cut-off values of PASP may vary, whereas a steep elevation in PASP during the early stages of exercise testing, even without reaching the cut-off value of 60 mmHg, may comprise another index of EIPH, which requires validation [26].

### 4.2. Cardiopulmonary Exercise Testing (CPET)

CPET is a diagnostic modality that is useful for the evaluation of unexplained dyspnea, when medical history, physical examination and common tests, such as electrocardiography, spirometry and cardiac or lung imaging, fail to identify a cause. A wide variety of diseases and pathological situations can be identified using CPET [27]. The exercise protocols used for CPET are similar to ESE, namely, the treadmill and ergo-cycling protocols. The preferred protocols use a gradual rise in workload during the exercise instead of a more steep-like protocol because the response to stress is analogous to the physiological response to real exercise. A facemask or a mouthpiece are used in order to collect data on ventilation and the respiratory gas exchange [28]. When the VE/VCO_2_ (VE: minute ventilation, VCO_2_: carbon dioxide output from the lungs) ratio is greater than 37 and PETCO_2_ (extrapolated end-tidal carbon dioxide tension) is below 30 mmHg at an anaerobic threshold, there is a great probability of EIPH [29,30]. Notably, measurements of PETCO_2_ below 20 mmHg are highly suggestive of EIPH and uncommon in other diseases [31]. CPET examination can be combined with an ESE providing not only functional but also structural data with a great diagnostic value for EIPH as well as a variety of pathological cardiac and lung conditions [25]. A great advantage of CPET is the simulation of real-life conditions by causing exertional dyspnea under controlled and monitored conditions and thereby identifying patients with mild abnormalities even at early stages of a cardiopulmonary disease. Moreover, CPET constitutes a reliable tool for the differential diagnosis of dyspnea whether the underlying cause is pulmonary, cardiac, ventilatory or metabolic [28]. Nevertheless, CPET provides a number of measurements, and their interpretation requires training and familiarization of the operator so the examination is reclaimed to its greatest.

Additionally, cardiac magnetic resonance (CMR) imaging during exercise is a recent diagnostic modality that is used only for research purposes so far and combines a bicycle featured in a CMR set-up [32]. Alternatively, a different protocol has been used with treadmill exercise outside the CMR set-up and immediate transport inside the CMR device for image acquisition but with limited application [33]. Overall, CMR enables the study of cardiac and pulmonary hemodynamics during exercise using a non-invasive technique. Cardiac index measurements with the CMR correlate with the direct calculation of cardiac output during RHC according to a previous study [34]. Compared to ESE, CMR provides a more objective and operator-independent measurement. Furthermore, CMR is a non-invasive technique and so the possible complications of central intravenous access are spared [31]. On the other hand, CMR is a more expensive modality, unfriendly for patients with claustrophobia, less reproducible for monitoring and requires specialized, highlyequipped centers. Its role seems promising in the future, providing that more studies are conducted for its efficacy evaluation.

### 4.3. Right Heart Catheterization (RHC) during Exercise

The first invasive pulmonary hemodynamic measurements during exercise in normal subjects were reported in the late 1940s. According to Fowler et al. (1969), the mPAP–flow relationship could be described using a linear approximation until the upper physiologically possible flows [35]. However, this correlation does not seem to be so simple and is difficult to describe precisely because of numerous co-founders, such as the type of exercise, age and the underlying cardiac and pulmonary comorbidities. RHC measurements, during exercise stress tests for the evaluation of dyspnea in subjects with normal resting pulmonary pressures, is a diagnostic technique that has been already used for several decades. As per the other two abovementioned diagnostic modalities, the invasive measurement of mPAP can be undertaken in a supine or upright position using an ergo-cycle or treadmill, respectively. At rest and during light exercise, mPAP is not affected by the exercise type (treadmill or cycling). However, during maximal exercise, the pulmonary pressures tend to be higher on the treadmill compared to the ergo-cycle.

Regarding the hemodynamic differences between upright and supine positions during exercise, the mPAP and cardiac output may be slightly decreased, and the heart rate increased in the former position [36]. Furthermore, supine or semisupine exercise is considered more sensitive because the venous return is maximized, and the detection of diastolic reserve impairments is more easily recognized. Another drawback of upright exercise is the creation of more artifacts and catheter whip [37]. The combination of RHC with CPET enables the combination of direct mPAP measurements with the analysis of the expired gas. However, the invasive nature of this diagnostic technique limits its repeated usage for EIPH detection or patients’ monitoring in daily clinical practice.

RHC during exercise tests is considered the gold standard for the evaluation of individuals with unexplained dyspnea [33]. A recent study investigated the correlation between the invasive measurement of mPAP and CPET calculations. It showed a statistically significant relationship between the respiratory parameters and mPAP at rest and at submaximal exercise, indicating a possible alternative method of EIPH assessment [38]. Another clinical study compared the pulmonary pressures measured by ESE and RHC during exercise, showing a correlation between them as long as the TR Doppler signal is of good quality [39]. A recent publication also investigating the association between ESE and RHC during exercise in patients with connective tissue disease proved that ESE is as reliable as an invasive method with regard to pulmonary pressure measurements. The addition of CPET to ESE examinations increased further their sensitivity [40].

Despite the aforementioned promising results, more studies need to be undertaken in order to evaluate the sensitivity and specificity of non-invasive modalities, such as ESE and CPET, as surrogate or adjunctive methods for EIPH diagnosis. A widely accepted strategy for EIPH diagnosis cannot be drawn. Based on previous evidence, it would be wise to start the investigation of EIPH with a non-invasive technique, either ESE or CPET, depending on the underlying clinical condition (e.g., ESE for MR), the local facilities and the experience of the center. In case the results of those tests are inconclusive, and the suspicion of EIPH remains high, then clinicians should proceed to the invasive RHC during exercise in order to confirm or reject EIPH.

## 5. Potential Clinical Applications

EIPH accurate diagnosis has been proposed as an essential component of the following clinical entities:

### 5.1. PH Diagnosis

The term PH diagnosis, included in the past EIPH, is as the elevation of mPAP above 30 mmHg in RHC during stress. However, in the 4th World Symposium on Pulmonary Hypertension (WSPH), this definition was abandoned due to a lack of evidence [41]. The latter decision was based partly on the lack of sufficient data and notably on a published systematic review that examined the mPAP values, via RHC, during rest and exercise. The results showed a heterogeneity of results with mPAP ranging below and above 30 mmHg during exercise in healthy individuals with an additional influence of age (above 50 years old) [32]. The entity of EIPH was also omitted from the 5th and 6th WSPH, which took place in 2013 and 2018, respectively, also because of limited data and the absence of validation [42,43].

EIPH is considered by several researchers as an early, precursor form of PAH, which may have a prognostic role in connective tissue disorders, such as systemic scleroderma [44,45,46,47,48]. Recently, clinical studies have investigated the relationship of EIPH with PAH development. A prospective study in a systemic sclerosis cohort reported the development of resting PH during the follow-up period, among individuals labeled with EIPH at baseline. The cut-off value for EIPH diagnosis was PASP > 50 mmHg, measured during ESE [49]. In another study recruiting patients with scleroderma, those with EIPH had increased mortality compared to patients with normal hemodynamic measurements during exercise [50]. The authors expressed the opinion that EIPH could be a precursor of pulmonary vascular pathology, affecting prognosis. Similarly, among patients with myelodysplastic syndrome the presence of EIPH indicates a higher risk of hospitalization [51].

Comparing ESE with RHC at rest, a recent study demonstrated a good diagnostic accuracy of exercise-induced PASP >50 mmHg, as it could unmask PH in selected patients with systemic sclerosis and baseline echocardiographic-based measurements of pulmonary pressure within the grey zone [52]. Regarding CPET, the intercept of ventilation and the VE/VCO2 (ventilation to carbon dioxide production relationship) slope have been significantly associated with mPAP measurements derived from RHC at rest in patients with suspected PH [53]. Similarly, a multicenter study of patients with scleroderma reported a positive correlation between the results of CPET and RHC at rest for the diagnosis of PH. In addition to this, peak VO2 (peak oxygen consumption) above 18.7 mL/min/kg could safely exclude PH, whereas VE/VCO2 may exert a prognostic value [50].

### 5.2. LV Valvular Diseases (Mitral Regurgitation—Primary/Secondary, Mitral Stenosis, Aortic Stenosis)

Mitral stenosis (MS): EIPH reflects exercise-triggered hemodynamic changes and can be an indicator of MS severity. De Castro et al. (2020) [54] showed an independent association of exercise-induced elevation in PASP with mean mitral gradient elevation, exaggerated right ventricular function, left atrial volume expansion and net atrioventricular compliance. In addition, a PASP rise at peak exercise was significantly correlated with an adverse outcome. The management of MS depends on symptoms and the echocardiographic assessment of its severity. However, those parameters may infrequently mismatch. In symptomatic patients, the development of symptoms on exertion may correlate with hemodynamic changes including PASP elevation [55]. Many so-called “asymptomatic” patients with severe MS appear with increased pulmonary pressures only during exercise but not at rest, which is determined mainly by the elevation in the mean mitral gradient. Identifying that subgroup of patients with severe MS with exercise-induced changes in PASP can be helpful in clinical decision-making. On exertion, a flow-dependent increase in the transmitral gradient, followed by an increase in LAP and PASP, is an important factor related to exercise intolerance in populations with significant MS. However, there are only a few small studies examining EIPH in MS [56,57,58]. The management of “asymptomatic” patients with nonsevere MS, developing symptoms and achieving high levels of PASP during exercise is not yet clearly defined. Unambiguously, more and larger studies are needed to determine the importance of EIPH in early surgical treatment and the outcome predictions of self-reported “asymptomatic” patients with severe MS.

Mitral regurgitation (MR): The European Society of Cardiology has reported that ESE is an important diagnostic tool to evaluate hemodynamic changes in patients with MR. In the recently updated guidelines, it was commented that ESE may be helpful for diagnosis and prognosis stratification when there is a mismatch between the symptoms and MR severity [59]. In asymptomatic patients with MR without LV abnormalities, ESE may unmask symptoms or identify a proportion of patients who are at higher risk of developing symptoms and who will benefit from early intervention. In a recent study of asymptomatic patients with primary MR, the early rise in PASP during exercise was a significant predictor of symptom occurrence and morbidity within one year, with an important proportion of them requiring surgical replacement [60]. A recent study in patients with MR following the watchful waiting strategy demonstrated poorer prognosis in those with EIPH, compared to those without EIPH [61]. The presence of EIPH in asymptomatic primary MR has been related to a worse prognosis [62]. In a study of 91 patients the presence of EIPH correlated with older age and a greater ratio of the mitral peak velocity of early filing to early diastolic mitral annular velocity during peak exercise [59]. Recent evidence has implicated that EIPH precedes symptom appearance in patients with severe MR [63]. Magne et al. (2013) showed that exercise-induced elevation in PASP was significantly associated with MR severity [55]. Moreover, the postoperative outcome in patients with MR depends on the presence and severity of preoperative symptoms. Postoperative prognosis worsens in symptomatic compared to asymptomatic patients prior to surgery [58,59]. The importance of early symptom detection and consequent intervention in asymptomatic patients is beyond any doubt. Therefore, ESE provides valuable prognostic information on the hemodynamic changes in MR, which could influence patient management. In asymptomatic patients with severe MR, EIPH may be an important determinant of surgery timing, while predicting outcomes. Nevertheless, the role of ESE and the calculated EIPH remain limited in the treatment strategy of MR patients.

Aortic stenosis (AS): As with other valvulopathies, the optimal timing of aortic valve replacement in asymptomatic patients with AS remains controversial [64]. Although the benefit of early surgical intervention in asymptomatic patients is still unproven, once symptoms develop the prognosis worsens [65]. In addition, elderly patients with severe AS tend to limit their physical activity to avoid symptoms, usually reporting a delayed symptom onset. It is quite challenging to distinguish the reason for exercise-induced dyspnea in these patients with other common comorbidities, including obesity, lung diseases, heart failure and physical deconditioning,. Therefore, an objective and feasible method to unmask symptoms and stratify patients according to their risk and the potential benefit of early surgical treatment is important. A significant proportion of asymptomatic patients with severe AS present EIPH [61]. In a recent study of asymptomatic AS patients with normal pulmonary pressure at baseline, a significant proportion of them developed high PASP during exercise. EIPH in this population was independently associated with higher morbidity and mortality at a 3-year follow up period [62]. The main determinants of EIPH in AS patients are male sex and resting PASP [66]. Thus, the early appearance of EIPH may be of clinical relevance, providing a valuable tool for the effective risk stratification of asymptomatic patients with AS.

HFrEF: PH at rest or during exercise is an important marker of a poor prognosis in patients with heart failure [67]. In patients with HFrEF, exercise-induced changes in PASP are variable and unrelated to resting PASP [68]. They seem to be associated with left heart dysfunction, including the lack of ventricle contractile reserve, intraventricular asynchrony and increased left atrium size. Left-sided heart diseases and heart failure are the most important causes of pulmonary hypertension, mainly due to elevated LAP. However, Verbrugge et al. [69] revealed an exercise-induced PVR increase with significant right ventricular dysfunction in this population. Another study [68] showed that PASP was significantly associated with ejection fraction and mitral effective regurgitation orifice (ERO) at peak exercise. The exercise testing of participants developing high exercise PASP was interrupted more frequently because of dyspnea. In this population, the worsening of MR and rising of the regurgitant volume and contractility dysfunction upon exertion were related to EIPH. The latter was attributed to backward pressures transmission following the enhanced functional MR and the inability of cardiac muscle to adapt. Myocardial asynchrony in these patients further exacerbates the phenomenon. On the other hand, the right ventricular systolic function is inversely correlated with exercise PASP, indicating right ventricular function worsening upon exertion [65].

Hypertrophic cardiomyopathy (HCM): A recently published study examined the prevalence and prognostic significance of EIPH in patients with HCM [70]. Those patients with additional EIPH had higher resting PASP, suggesting diastolic dysfunction and longer E wave deceleration time, a possible marker of impaired relaxation. Most importantly, the presence of EIPH was associated with adverse prognosis. Presumably, EIPH derives from elevated LV filling pressures, which could lead to the development of ventricular tachycardia and increased morbidity and mortality in these patients.

### 5.3. HFpEF (Patients with Unexplained Dyspnea)

Unexplained dyspnea is defined as a chronic, persistent dyspnea, where after basic laboratory and imaging investigations, the underlying etiology remains unknown [71]. Dyspnea can be a debilitating condition adversely affecting quality of life and prognosis. Frequently, physicians performing a long and meticulous work-up diagnosis in those patients conclude that a HFpEF diagnosis is the deeper cause of the unexplained dyspnea. Looking for the underlying causes in HFpEF, the most common reason seems to be the high LV filling pressures as a result of diastolic dysfunction [72,73,74]. However, previously published studies have doubted that mechanism [75,76]. According to a recent consensus paper published by the Heart Failure Association of the European Society of Cardiology during the diagnostic work up of dyspnea, a score (HFAPEEF) is used in order to assess the probability of HFpEF in patients with high suspicion but not initial definite diagnosis [25]. This score combines resting echocardiographic criteria and natriuretic peptides’ levels. In the case of patients with intermediate probability for HFpEF, diastolic stress echocardiography is recommended targeting values of E/e’ ratio above 15 and peak TR velocity above 3.4 m/s to set the HFpEF diagnosis.

Alternatively, invasive tests can be used when HFpEF is suspected. When normal measurements of LV filling pressures and mean pulmonary capillary wedge pressure (mPCWP) are invasively obtained at rest, but HFpEF suspicion persists, then RHC during exercise is recommended [73]. A PCPW above 25 mmHg can establish the diagnosis of HFpEF [77,78,79,80] as well as indicating a poorer prognosis [72,81,82]. Interestingly, a normal PCPW at rest and a steep increase during exercise is associated with a double mortality rate [82]. Taken together, an abnormal hemodynamic response to exercise may be considered an early form of HFpEF [73]. As a result, exercise testing may be useful for patients at risk or with masked HFpEF but without overt symptoms. Based on a recent meta-analysis, the add-on value of the E/e’ ratio calculation during ESE is significant and thereby increases the sensitivity of HFpEF diagnosis beyond resting measurements [83]. However, more studies need to be conducted to confirm the above results. Table 1 summarizes the abovementioned (Table 1).

## 6. Conclusions

EIPH refers to mPAP greater than 30 mmHg during exercise, and it is often an unidentified cause of exertional dyspnea in patients without obvious resting cardiac or respiratory pathology. Moreover, it is related to increased morbidity and mortality in patients with HFrEF or HFpEF, valvulopathies or respiratory diseases. The pathogenesis of EIPH is still not completely understood. Alterations in cardiac output, pulmonary vascular resistance and pressures in the left heart upon exertion may affect pulmonary pressures; hence, they are involved in EIPH. Although right heart catheterization remains the gold standard for EIPH diagnosis, because of its invasive nature, proposals have been made for the alternative use of ESE or CPET. The former is an easily conducted, cheap and repeatable technique. CPET offers a great variety of information, providing the capability of the differential diagnosis of unexplained dyspnea. Both techniques have significant advantages and disadvantages; however, more studies are deemed necessary to validate their results.

Regarding the clinical application of EIPH, it has recently raised by many scientists as a precursor stage of PH, which may trigger early initiation of the appropriate treatment. Similarly, the presence of EIPH, when there is a mismatch between symptoms and LV valvular disease severity, may objectively contribute to decision-making and earlier interventional therapy. Finally, EIPH may contribute to the diagnostic algorithm of HFpEF and HF patients’ risk stratification. The identification of normal pulmonary pressures at rest in patients with left-side heart diseases does not exclude cardiopulmonary dysfunction upon exertion. EIPH may be a valuable index of the severity, chronicity and prognosis in a wide spectrum of cardiopulmonary diseases.

## Figures and Tables

**Table 1:** Clinical applications of EIPH measurements.

Diagnostic Tool/Parameters	Diseases	Clinical Applications—“Strengths”	Limitations—“Weaknesses”
ESE (TRVmax PASP calculation)	PAH	(1) Predictor of PH in pts with systemic sclerosis with normal resting mPAP. (2) Higher risk of hospitalizations in pts with myelodysplastic syndrome. (3) Helpful for decision-making in asymptomatic pts with severe MS. (4) Helpful for diagnosis and risk stratification in asymptomatic pts with severe MR. (5) Helpful for diagnosis and risk stratification in asymptomatic pts with severe AS. (6) Index of worse prognosis in pts with HFrEF. (7) Index of worse prognosis in pts with HCM.	(1) PASP influenced by pulmonary function, exercise capacity and age. (2) Sometimes failure to get sufficient Doppler signal for TRVmax. (3) Inability to perform exercise test in older patients or those with orthopedic problems. (4) Need for cut-off value determination and validation.
	MS		
	MR		
	AS		
	HFrEF		
	HCM		
CPET (VE/VCO2, peak VO2, PETCO_2_)	PAH	(1) Good correlation with RHC for PAH diagnosis. (2) Normal values exclude PAH.	(1) Patients’ discomfort. (2) Complex interpretation. (3) Need for cut-off value determination and validation.
RHC during exercise (mPAP, wPAP)	MR	(1) Current gold standard method. (2) Helpful for diagnosis and prognosis of MR. (3) Helpful for diagnosis of HFpEF.	(1) Invasive technique. Potential complications and high facilities demand. (2) Not easily repeatable. (3) Artifacts and catheter whip during exercise.
	HFpEF		

AS, aortic stenosis; CPET, cardiopulmonary exercise testing; ESE, exercise–stress echocardiography; HCM, hypertrophic cardiomyopathy; MR, mitral regurgitation; HFpEF, heart failure preserved ejection fraction; HFrEF, heart failure reduced ejection fraction; mPAP, mean pulmonary artery pressure; MS, mitral stenosis; PASP, pulmonary artery systolic pressure; peak VO2, peak oxygen consumption; PETCO_2_, extrapolated end-tidal carbon dioxide tension; pts, patients; PAH, pulmonary artery hypertension; RHC; right heart catheterization; TRVmax, tricuspid regurgitation maximum velocity; VE/VCO2, ventilation to carbon dioxide production relationship; wPAP, wedge PAP.

## Data Availability

Not applicable.
